# Vaginal Dysbiotic Microbiome in Women With No Symptoms of Genital Infections

**DOI:** 10.3389/fcimb.2021.760459

**Published:** 2022-01-12

**Authors:** Rinku Pramanick, Neelam Nathani, Himangi Warke, Niranjan Mayadeo, Clara Aranha

**Affiliations:** ^1^ Department of Molecular Immunology and Microbiology, Indian Council of Medical Research (ICMR)-National Institute for Research in Reproductive and Child Health, Mumbai, India; ^2^ School of Applied Sciences & Technology (SAST-GTU), Gujarat Technological University, Ahmedabad, India; ^3^ Department of Obstetrics and Gynecology, King Edward Memorial Hospital and Seth Gordhandas Sunderdas Medical College, Mumbai, India

**Keywords:** microbiome & dysbiosis, vaginal microbiota (VMB), asymptomatic BV, Lactobacillus, bacterial vaginosis (BV)

## Abstract

The vaginal microbiome plays a critical role in determining the progression of female genital tract infections; however, little is known about the vaginal microbiota of Indian women. We aimed to investigate the vaginal microbial architecture of women with asymptomatic bacterial vaginosis (BV) (n=20) and normal microbiota (n=19). Microbial diversity was analyzed in vaginal swabs from regularly menstruating women (18-45yrs) by 16S rRNA V3-V4 amplicon (MiSeq Illumina) sequencing. Rarefaction analysis showed a higher number of species in normal flora compared to BV. Alpha diversity as measured by Pielou’s evenness revealed microbial diversity was significantly greater in BV samples than normal microbiota (p= 0.0165). Beta diversity comparison using UniFrac metrics indicated distinct microbial communities clustering between normal and BV flora. Firmicutes were the major phyla observed in vaginal specimens of normal microbiota whereas *Actinobacteria, Fusobacteria, Bacteroidetes* were significantly abundant in BV samples. Notably, the relative abundance of *Lactobacillus* was significantly high in normal microbiota. Conversely *Gardnerella*, *Sneathia*, *Prevotella*, *Atopobium*, *Ureaplasma*, *Dialister* significantly dominated dysbiotic microbiota. Relative frequency of Lactobacillus decreased significantly in BV (6%) as compared to normal microbiota (35.2%). *L. fermentum, L. gasseri, L. iners, L. jensenii, L. mucosae, L. ruminis, L. salivarius*, *L. coleohominis was* more exclusively present in normal microbiota. *L. iners* was detected from both the groups with a relative frequency of 50.4% and 17.2% in normal and BV microbiota respectively. Lefse analysis indicated *Atopobium vaginae, Sneathia amnii, Mycoplasma hominis Prevotella disiens* in the vaginal microbiota as a biomarker for dysbiosis and *L. jensenii* as a biomarker of a healthy microbiota*. Firmicutes* were negatively correlated to *Tenericutes, Actinobacteria, Bacteroidetes*, and *Fusobacteria*. Proteobacteria positively correlated to *Tenericute*s, and *Bacteroidetes* were shown to be positively correlated to *Fusobacteria.* Predicted functional analysis indicated differences in the functional profiles between BV and normal microbiota. Normal microbiota utilized pathways essential for phosphatidylglycerol biosynthesis I & II, peptidoglycan biosynthesis, geranylgeranyl diphosphate biosynthesis I, mevalonate pathway, CoA biosynthesis pathway I and pyrimidine nucleotide salvage; whereas BV bacteria had characteristic aromatic amino acid biosynthesis, pentose phosphate pathway, carbohydrate degradation. In conclusion, women with asymptomatic BV have vaginal microbiota significantly different than women with normal microbiota. Furthermore, the study provides insights into the vaginal microbial structure of Indian women that will enable us to explore the prospective candidates for restoring the vaginal microbiota.

## Introduction

Reproductive Tract Infections (RTIs) caused by bacteria, viruses or protozoa have a profound impact on the reproductive and sexual health of men and women. The consequences of sexually transmitted infections/reproductive Tract Infections (STIs/RTIs) for reproductive health include pelvic inflammatory disease (PID), infertility (in women and men), ectopic pregnancy, and adverse pregnancy outcomes including miscarriage, stillbirth, preterm birth, and congenital infection (Mullick et al., 2005; Odogwu et al., 2021). STIs and RTIs are also known to increase the risk of HIV transmission (Taha and Gray, 2000; van de Wijgert et al., 2008; Ward and Rönn, 2010).

The healthy vaginal microbiota is predominantly dominated by lactobacilli which help in maintaining a healthy vaginal microenvironment and protecting the host from urogenital infections. Women with a healthy vaginal microbiome, are known to mostly harbor *L. crispatus, L. gasseri, L. jensenii and L. iners* which have been reported previously (Nunn and Forney, 2016; Pramanick et al., 2019). Based on the predominance of these species the vaginal microbiota has been classified as CST I, II, III and V, respectively (Ravel et al., 2011).

The vaginal microbiome has been studied in pregnancy (Romero et al., 2014: Mehta et al., 2020), cervical cancer (Champer et al., 2018; Chambers et al., 2021; and infertility (Zhang et al., 2021). Alteration in the vaginal ecology during which the commensal lactobacilli are reduced and displaced by the anaerobic species of the genera *Gardnerella, Sneathia, Prevotella, Atopobium.* can lead to bacterial vaginosis. The vaginal dysbiosis not only affects the quality of life in women but could lead to poor reproductive sequelae in IVF patients (Haahr et al., 2016; Skafte-Holm et al., 2021), adverse pregnancy outcomes and increase risk of cancer (Yang et al., 2020). This change in the microbial ecology has been attributed to various factors such as sex hormones (Brotman et al., 2014), hormonal contraception (Achilles and Hillier, 2013), sexual practices, personal hygiene and diet (Lewis et al., 2017). Significant differences in the vaginal microbiome composition related to races/ethnicity have been reported (Peipert et al., 2008; Fettweis et al., 2014). However, in 15- 84% of apparently healthy women BV could be asymptomatic (Koumans et al., 2007; Pramanick et al., 2019). While many studies have characterized the vaginal microbiota of symptomatic cases of BV, asymptomatic BV remained uncharacterized.

India has a varied population and diversified ethnic composition. The limited vaginal microbiota studies on Indian women have focused only on lactobacilli (Garg et al., 2009; Madhivanan et al., 2015; Das Purkayastha et al., 2019). These studies have often reported contrasting observations. Furthermore, the microbiome of non-pregnant women of reproductive age in India remains unexplored. In this work, we have characterized the vaginal microbiota of a healthy and asymptomatic BV microbiota.

## Methods

### Study Participants and Recruitment

Vaginal swabs from 39 married, non-pregnant regularly menstruating women of the reproductive age group (18-45yrs) were collected for the study. The participants were recruited from the Gynecology and Obstetrics Out-Patients Department of King Edward Memorial Hospital and Seth Gordhandas Sunderdas Medical College, Mumbai. The study had the approval of the human ethics review board at the institute (Protocol Number 215/2012) and the collaborating institute (Protocol No EC/GOV-5/2012). Informed consent was obtained from all the participants. Inclusion criteria were women who were clinically healthy and in the age group of 18 - 45 years, had maintained sexual abstinence of at least 5 days, not using any vaginal hygienic products or lubricants, willing to get screened for STIs and other infections of the lower genital tract (vagina and cervix), have been diagnosed as normal with no infections of the lower genital tract based on the laboratory evidence, had not taken any antibiotics in the last six weeks, not menstruating and preferably 5 days following LMP, did not have any dysfunctional uterine bleeding or genital malignancy or genital prolapse. Pregnant women, women using hormonal contraceptives or OC’s, women with intrauterine devices, chronic drug intake, or with chronic medical illness were excluded from the study.

### Nugent Scoring and Amsel Criteria

One swab was used to assess vaginal health by Amsel’s criteria (Amsel et al., 1983) and Nugent scoring (Nugent et al., 1991). The second swab was used for DNA extraction for sequencing. Nugent scoring was based on the microscopic examination of different vaginal bacterial morphotypes and their enumeration on gram stained vaginal smears. Normal microbiota (score 0–3), intermediate (score 4–6), or BV microbiota (score 7–10) were the scores assigned by Nugent scoring. Amsel criteria were based on clinical symptoms and signs that include vaginal pH>4.5, the presence of clue cells (vaginal epithelial cells laden with coccobacilli) using wet mount microscopy, homogeneous white vaginal discharge and fishy odor of discharge (10% KOH amine test). A patient who satisfied three of these four criteria was diagnosed as positive for BV.

### Sample Processing and DNA Extraction

Genomic DNA was extracted according to the instruction on MO BIO Powersoil DNA extraction kit (Qiagen, Catalog No. 12888). The extracted DNA was quantified and purity was determined on the NanoDrop reader. The samples were stored at -20^0^C until further use.

### Amplicon Sequencing

The amplicons libraries were prepared using the Nextera XT Index kit (Illumina Inc.) as per the 16S metagenomic sequencing library preparation protocol. Primers for the amplification of the V3-V4 region of 16S rDNA were GCCTACGGGNGGCWGCAG and reverse ACTACHVGGGTATCTAATCC. The amplicon libraries were purified by AMPureXP beads and quantified using a Qubit fluorometer. The libraries were sequenced on MiSeq using 2x300 bp chemistry.

### Data Analysis and Statistics

Sequence read quality was assessed using Fastqc. Based on the observed quality, the reads were filtered out with the following parameters: all forward/reverse reads were trimmed by 10 bases from the left; from the right end, the reads were trimmed at length 280 for forward and length 220 for reverse. Reads were further pre-processed with DADA2 for denoising and eliminating chimera sequences and duplicates (**Supplementary Table 1**). The predicted ASVs were normalized by the minimum number of feature sequences in a sample from each of the study groups, respectively. Read pre-processing and taxonomic classification were performed in QIIME2 framework (Bolyen et al., 2019) using the pre-trained classifier for V3-V4 region of 16S rRNA genes of the SILVA database. Beta diversity was calculated with unweighted and weighted UniFrac metrics to compute the distribution of the samples of studied groups, viz., BV and Normal in Principal Coordinates Analysis (PCoA). Alpha diversity was estimated using the number of observed amplicon sequence variants (ASVs) using Pielou’s evenness index. The microbial taxonomy was studied for statistical differences (p < 0.05) between the studied groups and the core microbiota was compared using Microbiome Analyst (Dhariwal et al., 2017). Functional attributes corresponding to the observed microbiota were assessed using the q2-PICRUST plugin (Douglas et al., 2020). The statistical differences between the normal and BV were studied by performing Kruskal–Wallis test in the STAMP v2.1 software (Parks et al., 2014).

Linear discriminant analysis (LDA) Effect Size (LEfSe) method (Segata et al., 2011) was employed to predict biomarker species and functional attributes (LDA score cutoff value of 4) with significant differences in abundance between the two groups. Linear discriminant analysis (LDA) Effect Size (LEfSe) method was employed to predict biomarker species and functional attributes (LDA score cutoff value of 4) with significant differences in abundance between the two groups.

### Data Availability

The sequences were submitted in the NCBI database under the Bio project Accession No.: PRJNA674451. The names of the repository/repositories and accession number(s) can be found below: https://www.ncbi.nlm.nih.gov/bioproject/PRJNA674451.

## Results

For this study, we recruited 39 regularly menstruating women of the reproductive age group of 18-45yrs, who were not on antibiotics. Nugent scoring of the vaginal swabs revealed, 19 women had normal microbiota and 20 had BV. The recruited participant’s characteristics are summarized in **Table 1**. There was no significant difference in age and the menstrual phases of participants recruited for each group (**Table 1**). Due to its cosmopolitan nature the population of Mumbai is characterized by people from different parts of the country. About 15.38% (6) participants were nonresidents of Mumbai whereas 10.26% (4) have migrated in the last one year from sample collection.

**Table 1:** Characteristics of participants recruited for the study.

Groups	Study participants (N)	Vaginal pH		Age (yrs)		Menstrual phase		
		(mean ± SD)	P value	(mean ± SD)	P value	Follicular (n)	Luteal (n)	P value
**Normal**	19	3.85 ± 0.56	0.0001	30.79 ± 7.40	0.2402	11	8	0.5273
**BV**	20	5.46 ± 0.69		33.26 ± 5.42		9	12	
**Total**	39	_		_		20	20	

Two-tailed unpaired t-test was calculated to evaluate the statistical differences between normal and BV group for vaginal pH and age. Statistical differences between normal and BV group for menstrual phases were calculated using Fisher’s exact two tailed test. Results were statistically significant at a p-value less than 0.05.

The vaginal microbial profile of these women was characterized using Illumina MiSeq sequencing of the V3-V4 region of the 16s rRNA gene with an average read length of 301bp. A total of 7041,312 reads were generated from 39 swabs with an average of 352,006 sequences per sample. On average 92.38% of sequences had a Phred score of >20.

### Comparison of Vaginal Microbiota Between Healthy and Asymptomatic BV Samples

**Figure 1:**
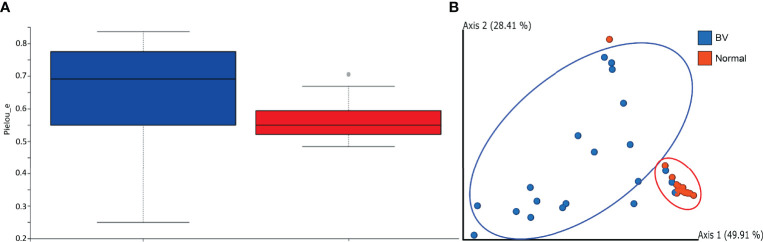
**(A)** Alpha-diversity represented by the Pielou evenness index in the BV and normal samples indicating significant difference (p-value = 0.0165) between the groups based on the Kruskal-Wallis test. The boxes represent the distributions of the alpha diversity index and show the median for each condition. Whiskers extend to the furthest data point. **(B)** PCoA generated using weighted Unifrac distance indicates distinct clustering of samples from each group. Each point corresponds to an individual sample. For each experimental group, an ellipse around the centroid is depicted. The first two components of the variance are represented by Plotting BV(blue) vs Normal (Red) samples with significant separation between the two groups. The first two components represent 78.32 percent of variance, individually depicted in parentheses next to Axis1 and Axis2.

The rarefaction analysis revealed a higher number of observed species in Normal microbiota compared to BV samples. However, the comparison difference revealed an FDR Adjusted P-value = 0.19 showing a less significant difference between Normal and BV samples. The rarefaction curve generated by the OTUs obtained from both groups indicated sufficient sequencing depth (**Supplementary Figure 1**). The alpha diversity measured by the Pielou evennessindex revealed women with normal microbiota had less diverse communities as compared to women with asymptomatic BV. As observed from **Figure 1A**, a significant (p=0.0165) difference exists between the community evenness of BV and Normal samples. Beta diversity measures the amount of dissimilarity between the samples. Beta diversity comparison using UniFrac metrics, used to measure differences in microbial abundances between the groups, demonstrated distinct microbial communities between normal and BV samples (p = 0.0127). The first two components of the PCoA explained 78.32% of the variations among the samples in axes 1 (49.95) and 2 (28.41%) respectively. The cluster corresponding to the normal microbiota showed variation along axis 1, from the BV population (**Figure 1B**).

**Figure 2:**
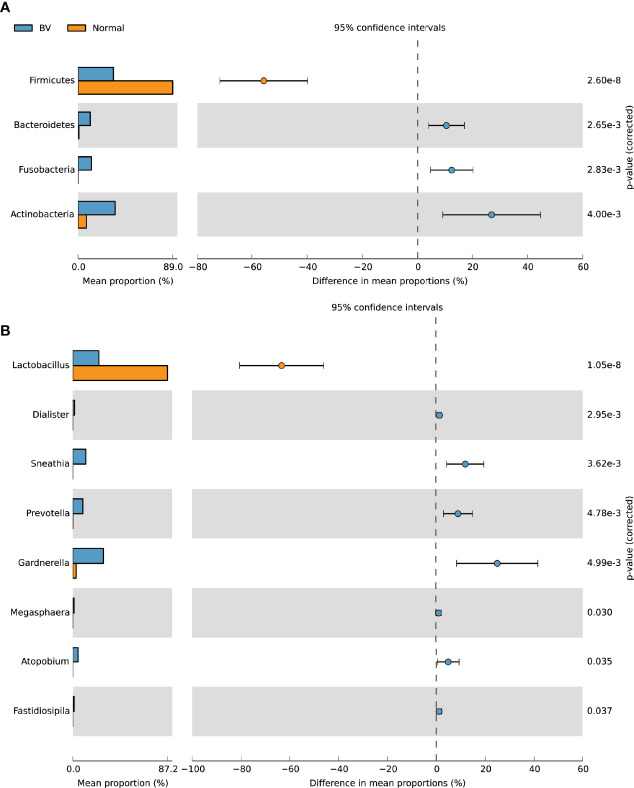
Relative abundance of bacteria at the **(A)** phylum and **(B)** genus level in the normal and BV groups as computed by Welch’s t-test using STAMP software. The middle shows the difference between proportions of abundance in the 95% confidence interval, and the value at the right is the P-value. P < 0.05 represents the significant difference between the two groups.

**Figure 3:**
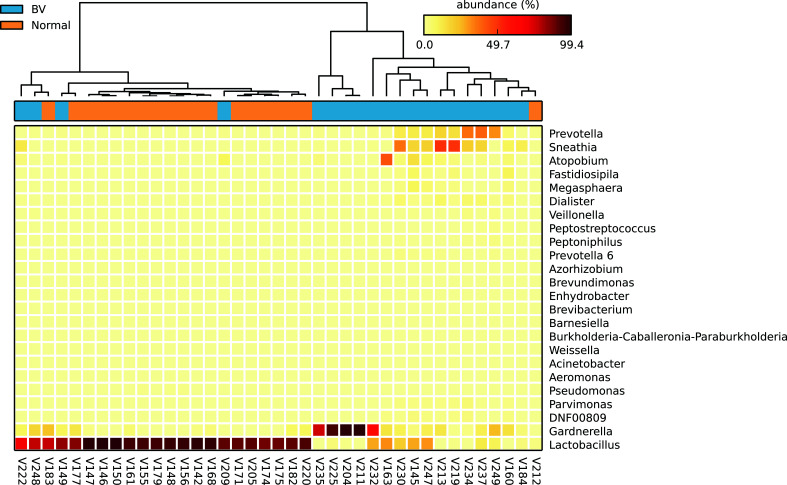
Genus level composition of the vaginal microbiota in normal and BV samples. The heatmap depicts the relative abundances of the most abundant genera. Each column represents one sample. Orange are samples with normal microbiota and Blue are BV samples.

*Firmicutes* were the most abundant phyla in normal microbiota whereas *Bacteroidetes, Actinobacteria, Fusobacteria* were significantly abundant in the asymptomatic BV group (**Figure 2A**). *Lactobacillus* was the predominant genus in normal microbiota whereas *Gardnerella*, *Sneathia*, *Prevotella*, *Atopobium*, significantly dominated the BV microbiota (**Figure 2B**). The heatmap in **Figure 3** depicts the abundance of the major genus present in each sample. The mean proportion of *Lactobacillus* and *Gardnerella* in normal microbiota was 87.2% and 3.1% as compared to 24% and 28.2% in BV samples respectively. *Sneathia* and *Atopobium* were absent in normal microbiota but constituted 12.1% and 4.9% in asymptomatic BV samples.

*Lactobacillus* was detected in all the samples of normal microbiota whereas it was completely missing from two samples with asymptomatic BV. *L. iners* (84.21%), (65%) and unidentified *Lactobacillus* spps. (84.21%), (80%) were the most frequently detected lactobacilli in both normal and BV samples respectively. Additionally, most of the women with normal microbiota harbored *L. jensenii* (36.84%) and *L. gasseri* (31.57%). Women with asymptomatic BV were frequently detected with *L. gasseri* (15%), *L. salivarius* (15%) and *L. fermentum* (15%). Other lactobacilli exclusively present in normal microbiota were *L. coleohominis* (10.52%) and *L. ruminis* (5.26%).

### Classification of Microbiota Based on Bacterial Markers

Taxonomic biomarker identification in each group was carried out using LefSe analysis. Discriminate analysis using LefSe showed *Lactobacillus jensenii*, *Comamonas, Weissella* were enriched in normal samples while *Atopobium vaginae*, *Sneathia amnii, Mycoplasma hominis* were enriched in BV samples (**Supplementary Figure 2**).

**Figure 4:**
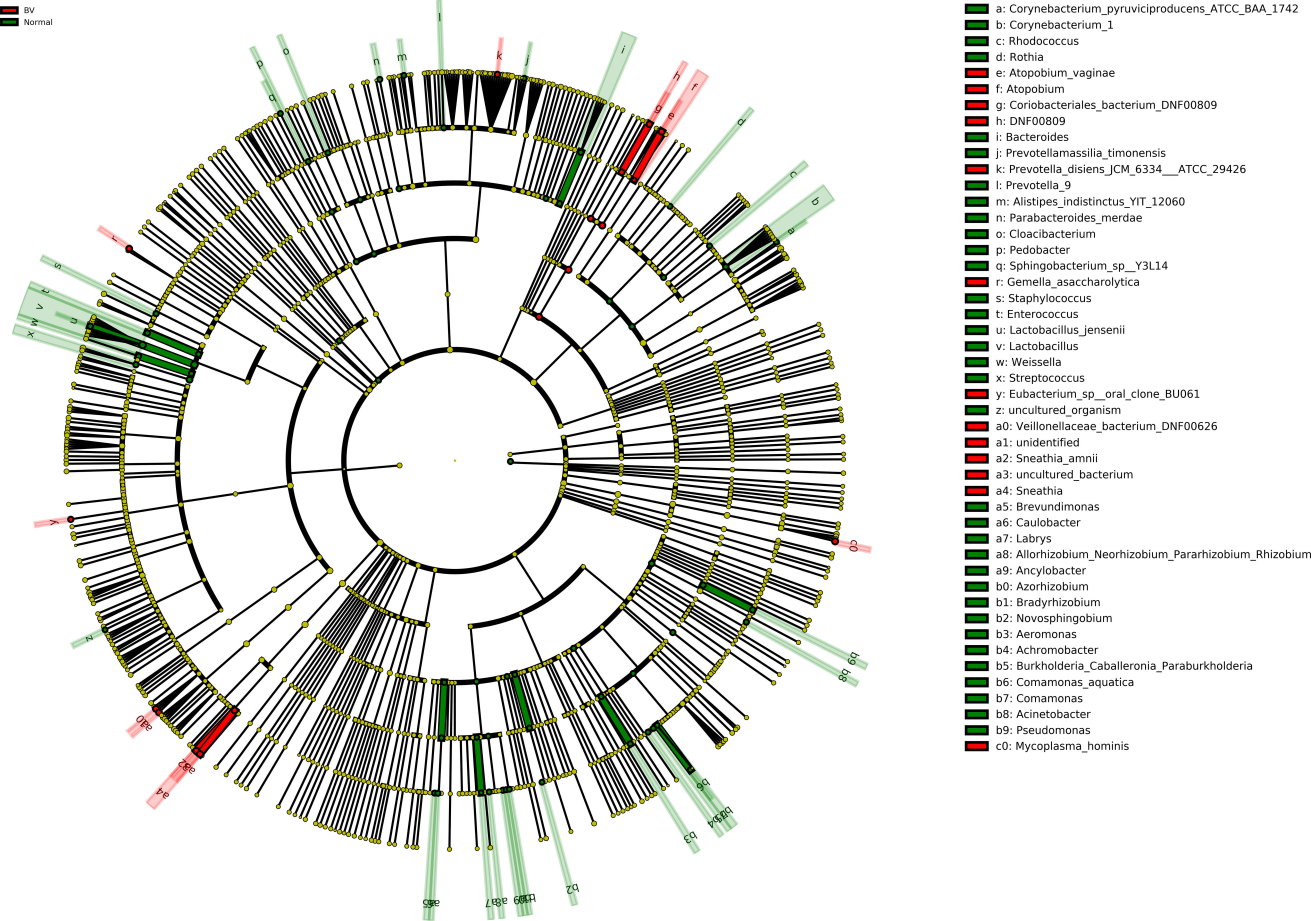
Cladogram representation of differentially abundant bacterial families detected using LEfSe. The cladogram diagram shows the microbial species with significant differences in the two groups. Red and green, indicate BV and normal microbiota groups respectively, with the species classification at the level of phylum, class, order, family, and genus shown from the outside to the inside. The red and green nodes in the phylogenetic tree represent microbial species that play an important role in the BV and normal microbiota groups, respectively. Yellow nodes represent species with no significant difference. Significantly abundant bacterial groups observed in the study are shown in the list on the right hand side.

Moreover, Cladogram represents differentially abundant genus and species (**Figure 4**). *Lactobacillus jensenii* was significantly abundant in normal microbiota as compared to asymptomatic BV. On the other hand, species such as *Atopobium vaginae, Coriobacteriales bacterium DNF00809, Sneathia amnii* were differentially elevated in asymptomatic BV samples.

### Correlation of Microbial Members of the Vaginal Ecology

**Figure 5:**
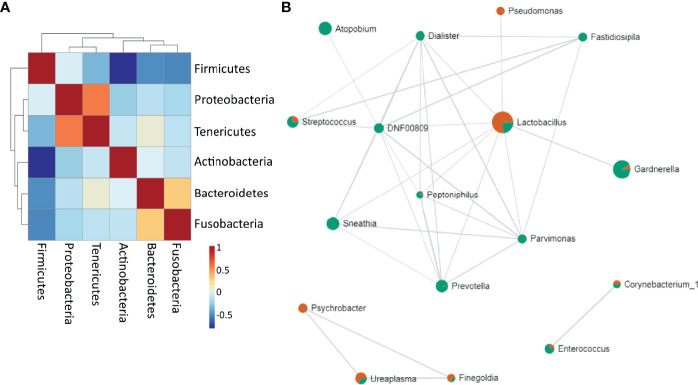
Bacterial correlation **(A)** at the phylum level. Positive correlations are represented as red and negative correlations as blue **(B)** Network of associations between the bacteria at the genus level. Red are samples with normal microbiota and green are BV samples. The nodes represent genera of bacteria; the edges represent the correlation coefficients between genera. Node size indicates the number of subjects in which the genus was seen.

Firmicutes were negatively correlated to Tenericutes, Actinobacteria, Bacteroidetes and Fusobacteria. Proteobacteria positively correlated to Tenericutes, and Bacteroidetes were shown to be positively correlated to Fusobacteria (**Figure 5A**). *Lactobacillus* highly negatively correlated to *Gardnerella* and other BV-related bacteria, but positively correlated to *Pseudomonas*. *Sneathia* positively correlated with *Enterococcus*, *Mycoplasma* and other pathogens such as *Dialister, Prevotella, Atopobium*, *Streptococcus* (**Figure 5B**) (**Supplementary Table 2**).

### Functional Profile of Vaginal Microbiota

**Figure 6:**
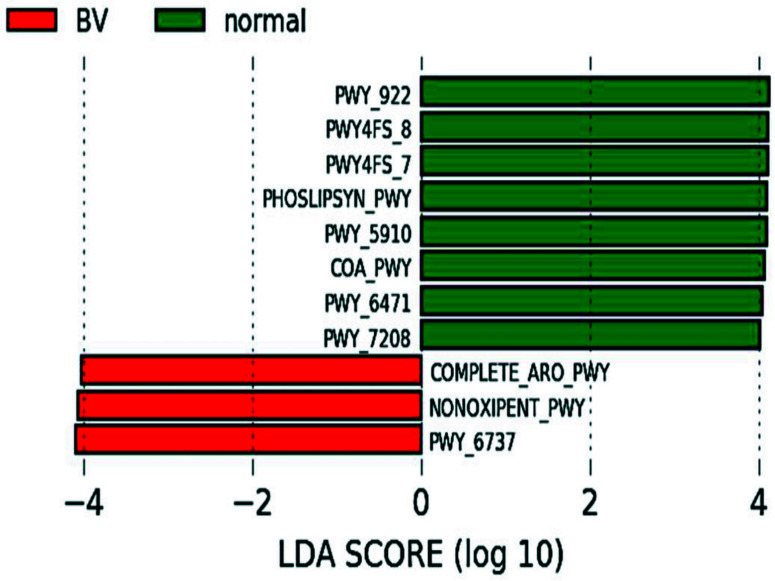
Predicted functional profiling: Differential pathway abundance predicted by PICRUSt between normal and BV group. Pathways (BioCyc IDs) with an LDA score of 4 and above in normal (Green) and BV (Red) samples are represented.

The normal microbiota was enriched in pathways involved in phosphatidylglycerol biosynthesis I & II, peptidoglycan biosynthesis, geranylgeranyl diphosphate biosynthesis I, mevalonate pathway, CoA biosynthesis pathway I and pyrimidine nucleotide salvage while the microbiota in BV women was enriched with aromatic amino acid biosynthesis, pentose phosphate pathway, carbohydrate degradation (**Figure 6** and **Table 2**).

**Table 2:** The enriched pathways in vaginal microbiota of Indian women.

Sample	Pathway code	Pathway name
Normal samples	PWY 922	Mevalonate pathway I
	PWY4FS-8	phosphatidylglycerol biosynthesis II
	PWY4FS-7	phosphatidylglycerol biosynthesis I
	Phoslipsyn-PWY	superpathway of phospholipid biosynthesis I
	PWY-5910	Geranylgeranyl diphosphate biosynthesis I
	Coa-pwy	coenzyme A biosynthesis I
	PWY-6471	peptidoglycan biosynthesis IV
	PWY-7208	pyrimidine nucleobases salvage
BV samples	Complete ARO PWY	Amino acid biosynthesis
	Nonoxipent pwy	Pentose phosphate pathway
	PWY 6737	carbohydrate degradation

## Discussion

*Lactobacillus* which is the keystone bacteria of vaginal microbiota was significantly present in the normal sample as compared to asymptomatic BV microbiota. In contrast to our observation, a study on Estonia women of reproductive age has reported lactobacilli dominance in healthy as well as asymptomatic BV women (Drell et al., 2013). *Lactobacillus*, *Comamonas, Weissella* were identified as the biomarkers for a healthy microbiota. *Lactobacillus, Leuconostoc, Weissella*, and *Streptococcus* have been commonly identified in vaginal samples of other populations (Jin et al., 2007). Along with *Lactobacillus* Weissella spp. have been isolated in fermented food items and feces (Ruiz-Moyano et al., 2011; Gomathi et al., 2014; Zhang et al., 2014; Kang et al., 2020; Pabari et al., 2020) and demonstrated to have probiotic and anti-inflammatory properties (Fatmawati et al, 2020). *Weissella* is another lactic acid bacterium known to produce H_2_O_2_ in the genital tract of women (Jin et al., 2007) and shown to be a potential probiotic for vaginal health (Lee, 2005). *Atopobium, Gardnerella, Sneathia* were the biomarker genus for asymptomatic BV. The taxonomic biomarker identified in BV samples was similar to previously reported in Chinese women (Ling et al., 2010). All the enriched genera in asymptomatic BV samples have been reported as vaginal pathogens in various studies and contribute to the sequelae of spontaneous abortions, preterm birth, infertility and cancer (Agarwal et al., 2020). The presence of dysbiotic microbiota in vagina of asymptomatic women addresses the need for the characterization of the microbiome in those women who may have spontaneous abortions or infertility problems or those who go in for *in vitro* fertilization and embryo transfer (IVF-ET) procedures (Koedooder et al., 2019; Kong et al., 2020; Wang et al., 2021). Our previous study on cultivable microbiota also reported a distinct microbial diversity of asymptomatic BV from the normal samples (Pramanick et al., 2019). The translational aspects of the vaginal microbiome and metabolome data could be exploited and based on the identification of specific biomarkers from BV and normal samples, new diagnostic point of care tests and treatment modalities could be developed.

The predicted key functional pathways in the normal microbiota were peptidoglycan biosynthesis, phosphatidylglycerol biosynthesis I and II, peptidoglycan biosynthesis IV and pyrimidine nucleotide salvage pathways. Increased cell wall organization and peptidoglycan biosynthesis in Lactobacillus dominated microbiomes have been associated with reduced FGT inflammation (Alisoltani et al., 2020) and in modulating the immune system (Song et al., 2018). Peptidoglycan biosynthesis IV and pyrimidine nucleotide salvage pathways have been further described during the proliferative phase of menstrual cycle and associated with increased bacterial proliferation (Chen et al., 2017). Our studies show that normal microbiota dominant in lactobacillus species showed mevalonate pathways. These strains are demonstrated to alleviate hyperlipidemia by modulating AMPK and downregulating cholesterol biosynthesis *via* the mevalonate pathway and Bloch pathway (Lew et al., 2020) and also increase the resilience of tissue cells to cholesterol-dependent cytolysins (Griffin et al., 2017). Geranylgeranyl diphosphate biosynthesis I (via mevalonate) forms geranylgeranyl diphosphate which is a crucial compound involved in the biosynthesis of a variety of terpenes and terpenoids, including central compounds such as ubiquinones and menaquinones. In addition, GGPP is also used in posttranslational modifications of proteins (geranylgeranylation) which is important for membrane adhesion as well as the function of some proteins (Jiang et al., 2017). Aromatic amino acid biosynthesis, carbohydrate degradation, pentose phosphate pathway that may cause preterm birth were enriched in asymptomatic BV women. Women who gave preterm birth had a vaginal microbiome enriched with the nonoxidative branch of the pentose phosphate pathway (Odogwu et al., 2021). Thus, functionally, many of the enriched BV pathways were also reflective of vaginal microbiota associated with preterm birth. Our findings of functionally enhanced pentose phosphate pathway in asymptomatic BV women have implications of the possibility of preterm deliveries in these women if not treated. Though the need to treat or not treat BV has been controversial our study supports that women with asymptomatic BV need to be treated to prevent adverse pregnancy outcomes.

From this study, we were able to establish the vaginal microbiome of non-pregnant Indian women for the first time. Our study shows that women with no genital symptoms of bacterial vaginosis may have a dysbiotic microbiome. The prevalence of asymptomatic BV in otherwise healthy women and functional pathways that may cause preterm birth highlights the need for further research on its pathogenesis. The presence of these bacteria in women with asymptomatic BV is worrisome. Hence it is imperative to maintain homeostasis to prevent any future episodes of infections.

## Data Availability Statement

The datasets presented in this study can be found in online repositories. The names of the repository/repositories and accession number(s) can be found below: https://www.ncbi.nlm.nih.gov/bioproject/PRJNA674451.

## Ethics Statement

The studies involving human participants were reviewed and approved by human ethics review board at the ICMR-National Institute for Research in Reproductive and Child Health (Protocol Number 215/2012) and the collaborating institute King Edward Hospital and Seth Gordhandas Sunderdas Medical CollegeGor(Protocol No EC/GOV-5/2012). The patients/participants provided their written informed consent to participate in this study.

## Author Contributions

RP and CA designed the study. NM and HW recruited the participants and carried out sample collection. RP carried out sample collection and its processing. NN performed the bioinformatic analysis. RP and CA interpreted the data. RP and CA wrote the paper. CA was involved in the acquisition of funding and review of the manuscript. All authors contributed to the article and approved the submitted version.

## Funding

This work was supported by the Indian Council of Medical Research (Grant number 5/7/726/2012-RHN).

## Conflict of Interest

The authors declare that the research was conducted in the absence of any commercial or financial relationships that could be construed as a potential conflict of interest.

## Publisher’s Note

All claims expressed in this article are solely those of the authors and do not necessarily represent those of their affiliated organizations, or those of the publisher, the editors and the reviewers. Any product that may be evaluated in this article, or claim that may be made by its manufacturer, is not guaranteed or endorsed by the publisher.

## Supplementary Material

Supplementary Figure 1 Rarefaction curves showing the number of observed species for samples belonging to BV and Normal condition at each rarefaction depth starting from 100 seqs/sample to 80000 seqs/sample

Supplementary Figure 2 Differentially abundant bacterial taxa between normal and BV microbiota by LEfSe analyses Significant bacterial genera were determined by the Kruskal-Wallis test (P<0.05) with LDA score greater than 2. Normal and BV microbiota are represented by green and red bars respectively.

Supplementary Table 1 Sequence statistics of the samples after deionizing by DADA2

Supplementary Table 2 Pearson correlation coefficients and P-values between bacterial taxa.
