# Supplementary figures and images for: Vaginal Dysbiotic Microbiome in Women With No Symptoms of Genital Infections
Source: Front Cell Infect Microbiol. 2022 Jan 12;11:760459. doi: 10.3389/fcimb.2021.760459 (PMC8790106; doi:10.3389/fcimb.2021.760459)

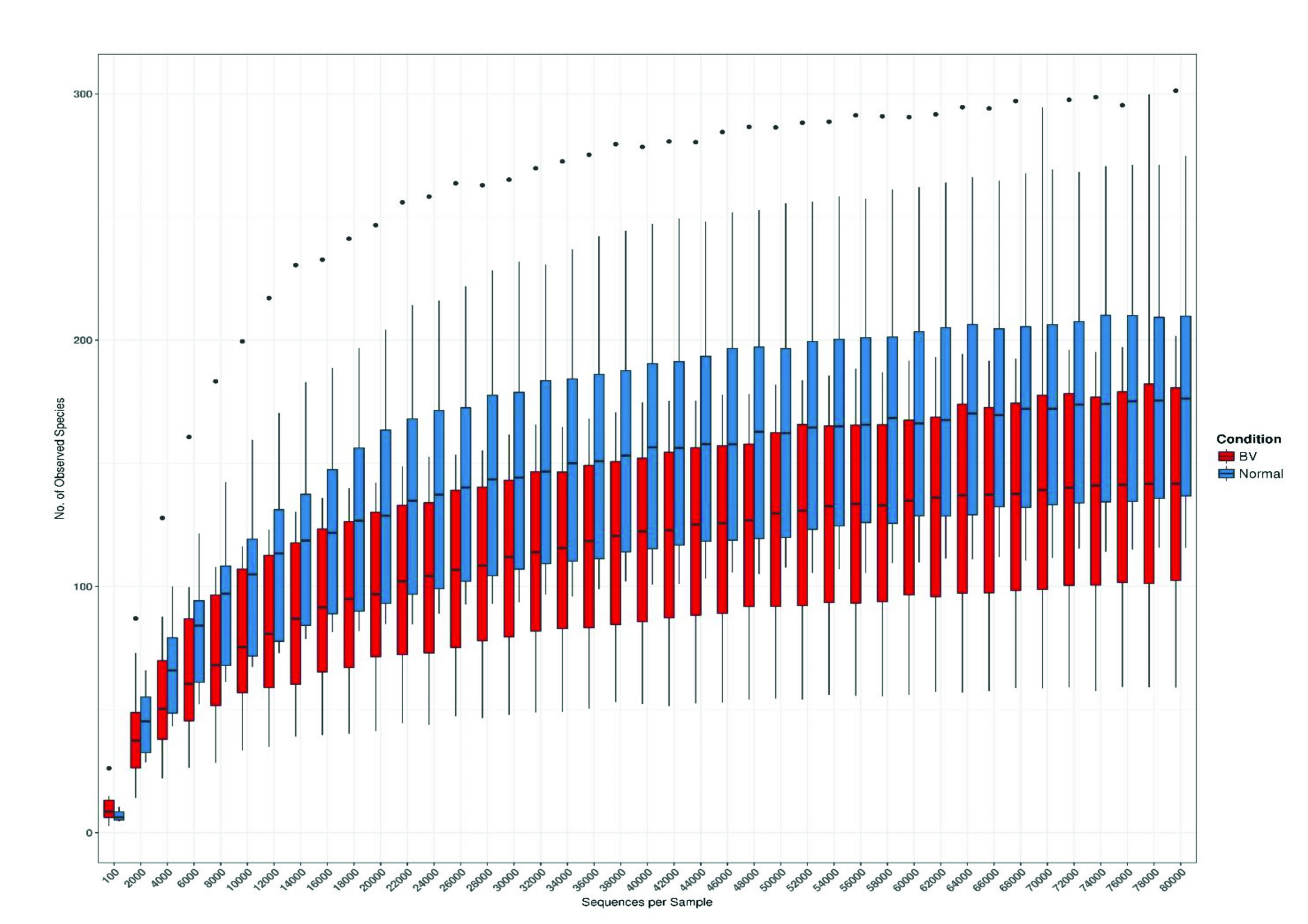

Supplement: Supplementary Figure 1 — Rarefaction curves showing the number of observed species for samples belonging to BV and Normal condition at each rarefaction depth starting from 100 seqs/sample to 80000 seqs/sample [file Image_1.tif]
