# Supplementary material for: Vaginal Dysbiotic Microbiome in Women With No Symptoms of Genital Infections
Source: Front Cell Infect Microbiol. 2022 Jan 12;11:760459. doi: 10.3389/fcimb.2021.760459 (PMC8790106; doi:10.3389/fcimb.2021.760459)
Supplement: Supplementary Table 2 — Pearson correlation coefficients and P-values between bacterial taxa. [file Table_2.docx]

| **Taxon1** | **Taxon2** | **Correlation** | **P.value** |
| --- | --- | --- | --- |
| Atopobium | DNF00809 | 0.3572 | 0.0256 |
| Corynebacterium_1 | Enterococcus | 0.8498 | 0 |
| Dialister | DNF00809 | 0.6904 | 0 |
| Dialister | Fastidiosipila | 0.5582 | 2.00E-04 |
| Dialister | Lactobacillus | -0.4656 | 0.0028 |
| Dialister | Parvimonas | 0.7805 | 0 |
| Dialister | Peptoniphilus | 0.542 | 4.00E-04 |
| Dialister | Prevotella | 0.6081 | 0 |
| Dialister | Sneathia | 0.4903 | 0.0015 |
| Dialister | Streptococcus | 0.3567 | 0.0258 |
| DNF00809 | Fastidiosipila | 0.8169 | 0 |
| DNF00809 | Lactobacillus | -0.4504 | 0.004 |
| DNF00809 | Parvimonas | 0.8104 | 0 |
| DNF00809 | Peptoniphilus | 0.3566 | 0.0259 |
| DNF00809 | Prevotella | 0.4445 | 0.0046 |
| DNF00809 | Sneathia | 0.4687 | 0.0026 |
| DNF00809 | Streptococcus | 0.545 | 3.00E-04 |
| Fastidiosipila | Parvimonas | 0.4814 | 0.0019 |
| Fastidiosipila | Streptococcus | 0.7533 | 0 |
| Finegoldia | Psychrobacter | 0.7026 | 0 |
| Finegoldia | Ureaplasma | 0.9721 | 0 |
| Gardnerella | Lactobacillus | -0.6597 | 0 |
| Lactobacillus | Parvimonas | -0.4538 | 0.0037 |
| Lactobacillus | Peptoniphilus | -0.3994 | 0.0118 |
| Lactobacillus | Prevotella | -0.4812 | 0.0019 |
| Lactobacillus | Pseudomonas | 0.4331 | 0.0059 |
| Lactobacillus | Sneathia | -0.447 | 0.0043 |
| Parvimonas | Peptoniphilus | 0.549 | 3.00E-04 |
| Parvimonas | Prevotella | 0.654 | 0 |
| Parvimonas | Sneathia | 0.6948 | 0 |
| Peptoniphilus | Prevotella | 0.9373 | 0 |
| Prevotella | Sneathia | 0.3748 | 0.0187 |
| Psychrobacter | Ureaplasma | 0.7068 | 0 |
